# Multiple Organ Involvement with Hydatid Cysts

**Published:** 2010-06

**Authors:** F Sabouni, F Ferdosian, S Mamishi, F Nejat, M Monnajemzadeh, N Rezaei

**Affiliations:** 1 Pediatric Infectious Diseases Research Center and Dept. of Pediatrics, Children's Medical Center, Tehran University of Medical Sciences, Tehran, Iran; 2 Division of Pediatric Neurosurgery, Children's Medical Center, Tehran University of Medical Sciences, Tehran, Iran; 3 Division of Pathology, Children's Medical Center, Tehran University of Medical Sciences, Tehran, Iran; 4 Research Group for Immunodeficiencies, Pediatrics Center of Excellence, Children's Medical Center, Tehran University of Medical Sciences, Tehran, Iran

**Keywords:** Hydatid cyst, *Echinococcus granulosus*, Multiple organ hydatidosis, Disseminated echinococcosis, Iran

## Abstract

Hydatid disease is the most common infections worldwide, but it rarely involves multiple organs. Herein, a 12-year-old boy is presented, who was admitted to Children's Medical Center, Tehran University of Medical Sciences, Tehran, Iran with symptoms of irritability, sleepless, and weakness of the extremities. Patient's brain computed tomography (CT) scan with contrast media showed large multilocular cystic lesions in right temporal lobe associated with two other smaller similar cystic lesions in centrum semiovale bilaterally. Abdominal sonography revealed intestinal mesenteric and a cardiac cyst. Abdomino-pelvic CT scan showed a cyst medial to the cecum and a cortical cyst in the left kidney as well as a heart cyst. The echocardiography confirmed hydatid cysts at apical and interventricular septum. Serology test was positive for hydatid cyst. Albendazole and praziquantel were started for the patient immediately and right temporal lobe lesions were removed via neurosurgery intervention. After one month, cardiac and mesenteric cysts were operated during two separate surgeries. Pathologic findings of all cysts were compatible with hydatid cyst. Cystic hydatidosis should be suspected in any cystic mass, whilst prompt diagnosis and appropriate treatments are the keys in management of affected patients.

## Introduction

Echinococcosis, also named as hydatid disease or hydatidosis, is the most common infections in human worldwide (1–3). Although hydatid disease is widely endemic in the Middle Eastern countries, including Iran (3), it is a rare disease during childhood. The hydatid cysts of *Echinococcus granulosus* tend to involve the liver (50–70 percent) or lung (20–30 percent), but may be found in any other organ, including brain, heart, and bones (<10 percent). Symptoms of disease are often absent, whilst infection is detected only incidentally by imaging studies in many cases. Common human organs for cysts are the liver, lung, and brain (1–3). However, cardiac with brain echinococcosis are rare parasitic diseases, almost in 0.5–2 percent of all cases (1–6). Hydatid cysts rupture into left-sided cardiac chambers may cause systemic emboli, and if ruptured into right-sided cardiac chambers may cause pulmonary emboli (4–7). Kidney involvement in echinococcosis is also very rare with only 2–3 percent of all patients (8).

We report here a 12-year-old boy with multiple organ involvement hydatid cysts, including brain, heart, intestine, and kidney, which was presented with the only neurological symptoms rather than other organs involvement.

## Case Report

**Fig. 1:**
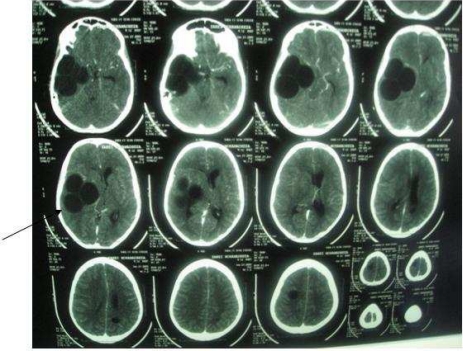
Brain computed tomography scan shows large multilocular cystic lesions about 70×65 mm in right temporal lobe with some mass effect over the adjacent structures.

**Fig. 2:**
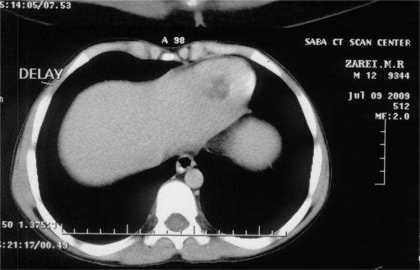
Abdominopelvic computerized tomography demonstrates a hydatid cyst of the heart with thickening of interventricular septum

**Fig. 3:**
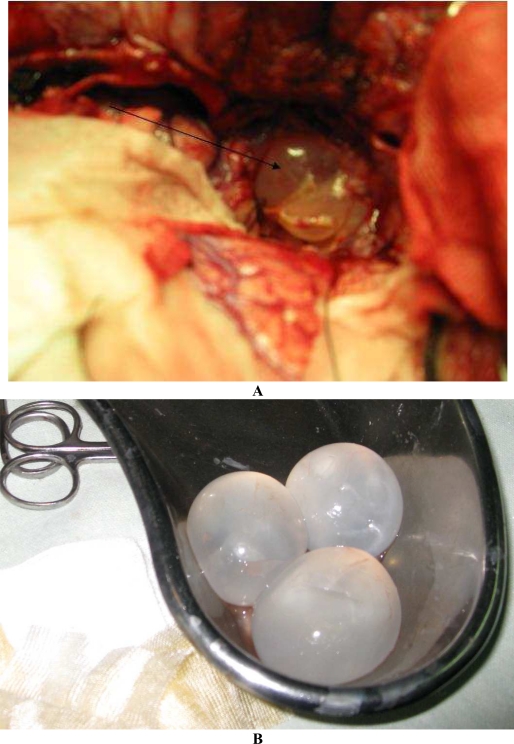
A. Brain hydatid cysts in the right temporal lobe (Operation field). B. Three extracted intact hydatid cysts of brain (Post operation)

A 12-year-old boy was admitted to the Children's Medical Center, the referral center in Tehran-Iran, with symptoms of irritability, sleepless, and weakness in the left extremities. He was living in a town without an exposure to animals. He had history of headache and frequent vomiting since 3 weeks before admission and medial deviation of right eye for 1 week. Physical examination revealed bilateral papilledema, left hemiparesis, left central facial nerve palsy and an increase deep tendon re?exes. Routine blood tests, erythrocyte sedimentation test, and urinalysis were normal. Chest roentgenogram was normal. Findings of a brain computed tomography (CT) scan with contrast media showed large multilocular cystic lesion about 70×65 mm in right temporal lobe with some mass effect over the adjacent structures and ipsilateral ventricle (Fig. 1). Two other smaller similar cystic lesions were also noted in centrum semiovale bilaterally. There was no evidence of pathologic enhancement or calcification around the lesions. Ultrasonogram (US) of the abdomen and abdominopelvic CT scan demonstrated thickening of interventricular septum of the heart with cystic attenuation (Fig. 2) and a cortical cyst about 10 mm in the left kidney and one cyst about 20 mm medial to the cecum noticed. Echocardiography revealed good left ventricle function with ejection fraction 69% and a cyst at apical and interventricular septum with a size of 2.2×2.7 cm. The result of a serum antibody test for hydatidosis was positive. At first step, albendazol and praziquantel was prescribed; and intervention was made by pediatric neurosurgeon to remove the brain cysts without rupture (Fig. 3). In the second step, the cardiac cyst that contained many daughter cysts was operated by pediatric cardiac surgeon. After one month, the intestinal cyst was removed by pediatric surgeon. Albendazole and praziquantel were continued as medical therapy.

## Discussion

Although hydatid disease is widely endemic in the Middle Eastern countries, such as Iran (3, 9), there are only few reports on clinical characteristics and appropriate treatment of the disease in childhood (10). Indeed multiple organ involvement with hydatid cysts rarely occurs (1).

Herein, we report an Iranian 12-year-old boy with disseminated cystic echinococcosis in the brain, heart, kidney, and intestinal mesenter without involvement of lung and liver. It is estimated that the central nervous system could be involved in 50–75% of cases with hydatid disease in children. The cysts could be enlarged slowly and progressively and the patients with cerebral involvement have a good condition without neurological deficits, in spite of the large size of the cyst in most cases, which is compatible with our patient's history.

Cerebral hydatid cysts may be either primary or secondary. Primary cysts, which are the most common type and are usually solitary, can be caused because of direct seeding of multiple larvae from blood stream into the central nervous system tissues. Secondary hydatid cysts of the brain may occur spontaneous, hematogenous, traumatic, or due to surgical rupture of a primary cerebral or cardiac cyst (1, 4, 7). Primary involvement of the kidney without involvement of the liver and lungs is also considered a very rare condition. Hydatid cysts may be found in only 10–20 percent of all cases with renal hydatidosis and are usually microscopic (4, 5, 8). Radiological imaging can assist the clinicians in making the precise diagnosis of hydatid disease. We detected the brain cysts in our patient by brain CT scan. The tests for diagnosis of hydatidosis include detection of IgE and IgG by Western Blot and molecular analysis of the excised tissues. Sequencing of the DNA polymerase chain reaction of contents from the cyst could also be performed as more specific test (11).

Management of disseminated echinococcal disease is complex, which requires a multidisciplinary approach (12). Hydatid disease should be treated with either albendazole and surgery or albendazole and PAIR (Puncture, Aspiration, Injection of scolicidal agent, and Reaspiration) to extract the cyst without any complications (1, 2, 4). In our case, the multiple organ cysts were removed surgically without any problem and medical therapy was continued by albendazole and praziquantel without significant complication. It has been suggested that albendazole can decrease recurrence of disease; meanwhile it can be used in treatment of inoperable cysts. As of high frequency of recurrent hydatid disease after treatment with only surgery or only albendazole, multidisciplinary approach is recommended and continuing medical treatment for at least 3 months postoperatively is also suggested (12). In patients with multiorgan hydatid cysts, surgical removal of all cysts at the same time is recommended. Treatment with mebendazole or albendazole have also very effective results in patients with multiple or inoperable cysts (10). Albendazole, an oral benzimidazole antihelmintic agent, is a drug of choice for the medical therapy of echinococcal disease (13), which should be used in patients with inoperable primary cyst and those with peritoneal hydatid cysts. It can prevent to repeat of echinococcal infection secondary to spillage of cyst's contents during surgery or drainage of cyst. The dose of drug is 10 to 15 mg/kg/d in 2 divided doses given in cycles of 4 weeks, without drug therapy for 2 weeks. This regimen is continued for several cycles depending on the severity of disease or the improvement of patients (3–13). Mebendazole could also be considered as an alternative drug, but it is less effective because of poor absorption. Praziquantel, an isoquinoline anthelmintic, is a potent protoscolicidal agent in vitro (10–13). The usage of albendazole with praziquantel together may be more effective than albendazole alone. The condition of our patient is very well. It will be maintained on albendazole, praziquantel, close medical follow-up, and serial imaging studies.

The PAIR procedure is associated with greater efficacy and lower rates of complications than conventional surgery for the treatment of liver hydatid cysts. The procedure also has been used successfully in the treatment of other organs such as lungs and renal hydatid cysts (10–14). In the PAIR, metronidazole could also be used with a similar effect of hypertonic saline on the membrane of hydatid cyst (14). We started albendazole, praziquantel, and metronidazole for our patient before operation.

In conclusion, in endemic areas, cystic hydatidosis should be considered for the diagnosis of a patient with cystic mass lesion. History and clinical data, the epidemiology of the disease, the imaging and immunological tests could assist the physicians to make definite diagnosis. Echinococcosis should be treated medically with albendazole, praziquantel, and either surgery or PAIR to remove the cyst successfully without any complication.
